# A Rare Case of Primary Malignant Melanoma of the Esophagus Presenting as Dysphagia

**DOI:** 10.14309/crj.0000000000001405

**Published:** 2024-07-18

**Authors:** Ahtshamullah Chaudhry, Gabriel Buluku, Jawad Noor, M. Chaudhari, Challa Suryanarayana, Steven Bigler, Stewart Boyd, Makau P. Lee

**Affiliations:** 1 Department of Internal Medicine, St Dominic-Jackson Memorial Hospital, Jackson, MS; 2 Department of Gastroenterology, Howard University Hospital, Washington, DC; 3 Department of Pathology, Baptist Hospital, Jackson, MS; 4 Department of Gastroenterology, University of Mississippi Medical Center, Jackson, MS

**Keywords:** esophageal melanoma, dysphagia, esophageal bleeding

## Abstract

Melanoma is one of the most notorious tumors due to its appearance in unusual locations. The most frequent site is the skin; however, it can sporadically develop as a primary tumor in the esophagus. However, as symptoms appear later, if the primary site is in the gastrointestinal system, it is frequently detected at the metastatic stage. We hereby describe a case of primary malignant melanoma of the esophagus that presented due to dysphagia with solid food and on further workup, found to be at the metastatic stage.

## INTRODUCTION

Malignant melanoma is a neoplasm that arises from epidermal melanocytes and occurs commonly in the skin (95%), with the eye being the second most common location, while <1% are found arising from mucosal surfaces.^1^ Primary malignant melanoma of the esophagus (PMME) is extremely rare, predominantly affecting male patients aged 50–70 years and tends to be aggressive.^2^ Primary melanomas of mucosal surfaces remain clinically silent for a long time, often detected in late stages with metastasis and has average life expectancy of <15 months at the time of diagnosis.^3^ We present a case of PMME in a middle-aged man presenting with dysphagia and weight loss.

## CASE REPORT

A 73-year-old White man with a history of type 2 diabetes mellitus, hypertension, and dyslipidemia presented to outpatient clinic complaining of a metallic taste, dyspepsia, dysphagia to solid food, postprandial nausea and regurgitation of undigested food, and significant weight loss for several months.

**Figure 1.**
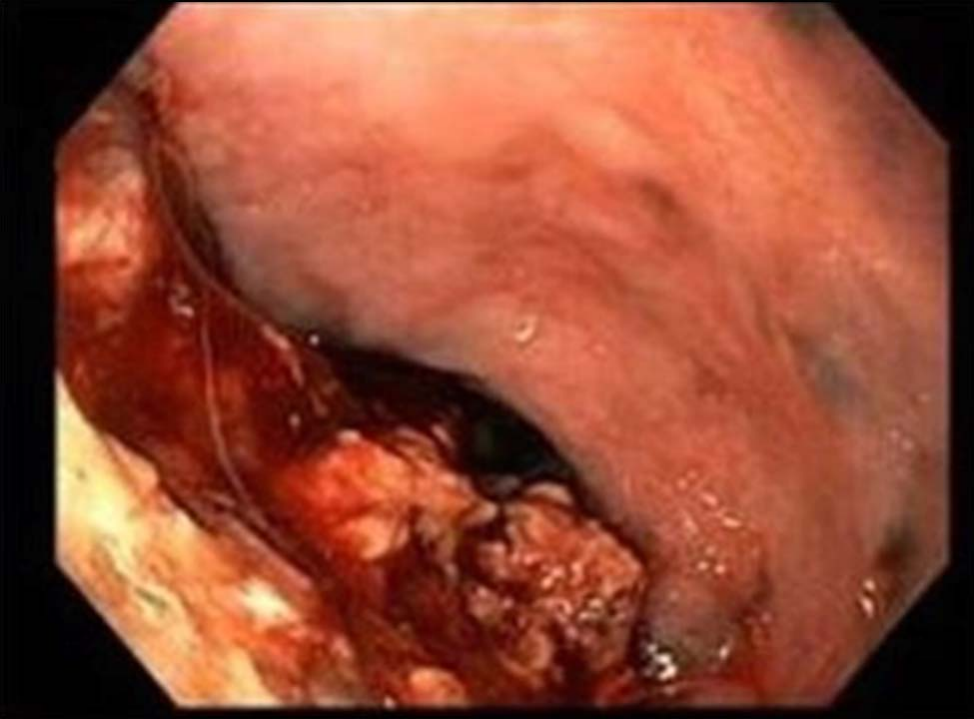
Endoscopic pictures depict an ulcerating mass at the lower gastroesophageal junction.

At upper endoscopy (EGD), the patient was noted to have a food bolus impacted in the distal esophagus, which was removed. Upon removal of the food bolus, a black ulcerated 3 cm mass partially obstructing the gastroesophageal junction was discovered; multiple biopsies of this mass lesion were obtained (Figure 1). The patient was hospitalized after EGD due to excessive bleeding, resulting from endoscopic biopsies and esophageal obstruction secondary to the obstructing tumor.

**Figure 2.**
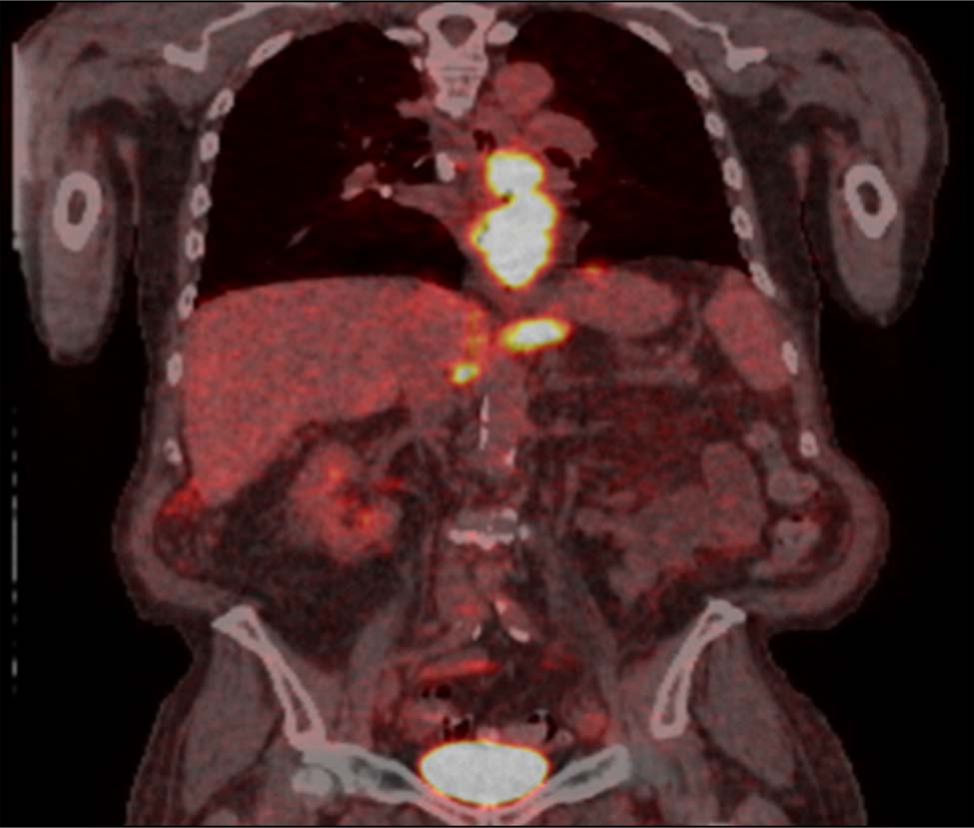
A positron emission tomography scan showed a 3.4 × 2.8 cm mass extending from distal esophagus to gastroesophageal junction with metastatic lymph node activity.

**Figure 3.**
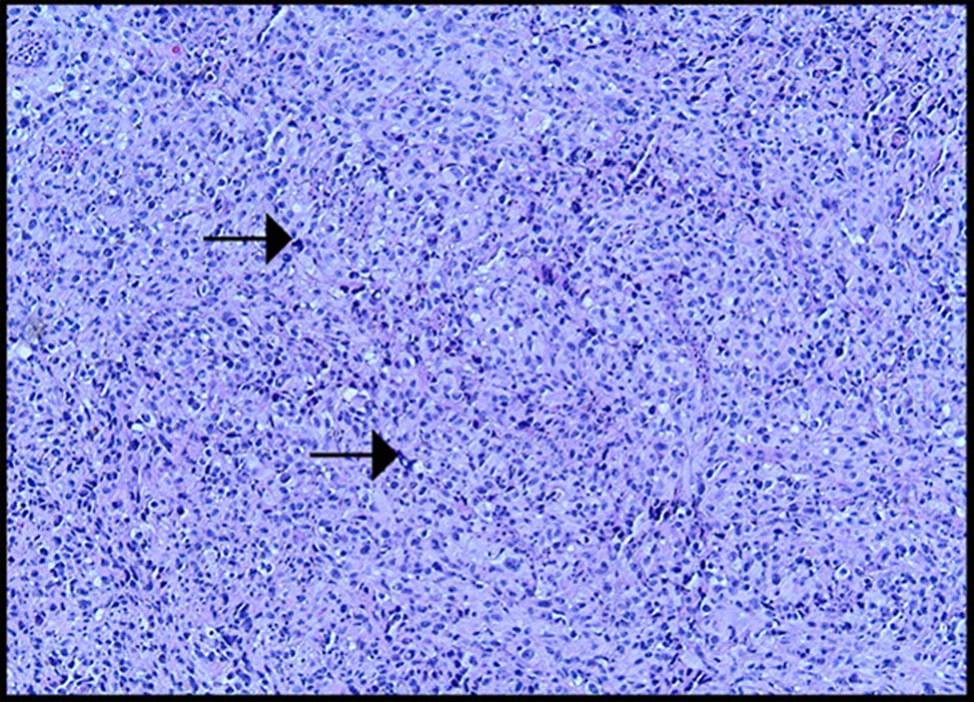
The tumor cells are arranged in sheets and show severe nuclear atypia (arrows), magnification 10×.

**Figure 4.**
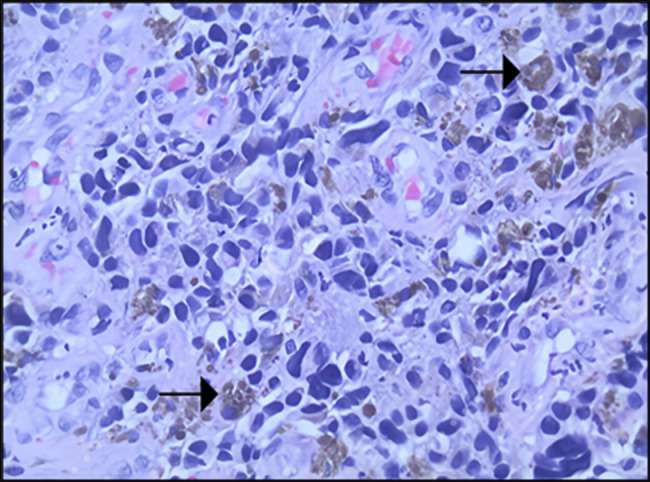
Higher magnification (40×) shows scattered brown pigment (melanin, arrows) among atypical tumor cells.

**Figure 5.**
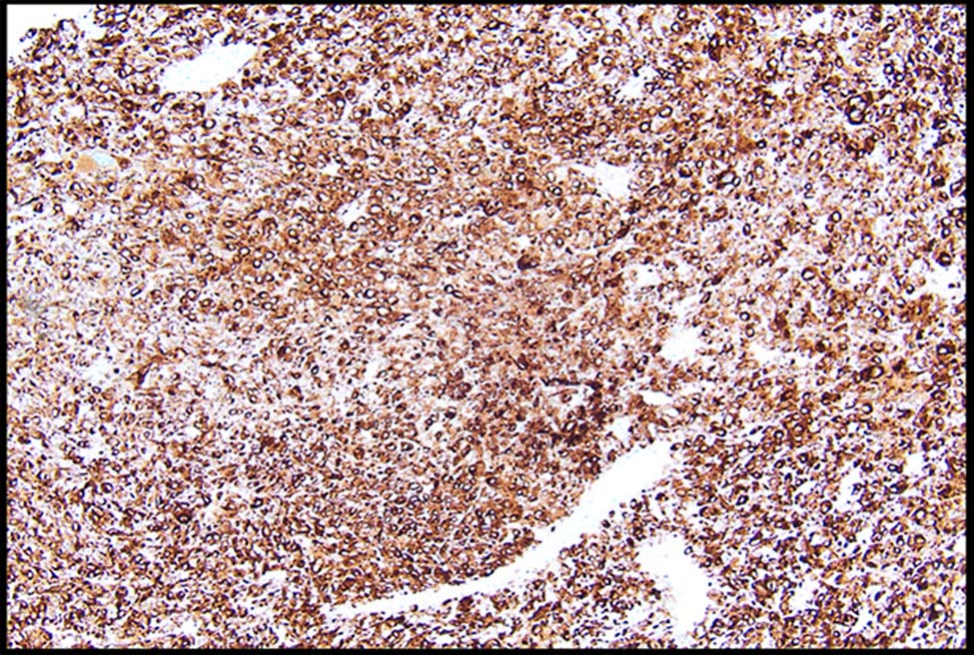
Tumor cells are positive for melanocytic marker MART-1, 10×.

A computed tomography of the chest, abdomen, and pelvis showed diffuse intra-abdominal adenopathy, suggestive of metastatic disease. A positron emission tomography scan showed a 3.4 × 2.8 cm mass extending from distal esophagus to gastroesophageal junction, with metabolic activity at paraoesophageal, gastrohepatic ligament and upper surface of the liver (Figure 2). Histologic examination of the biopsy specimen showed PMME, which was confirmed by comprehensive molecular genetic testing to distinguish between primary and metastatic cancer which was positive for melanoma-associated antigen recognized by T Cells (MART-1) (same as Melan-A) and negative for Pankeratin AE1/AE3, CK 7, CK 20, CK 5/6, p53, MOC-31, TTF-1, CDX-2, BRAF, KIT, and NRAS (Figures 3–5). The patient was discharged home after percutaneous endoscopic gastrostomy tube placement for nutritional support, with outpatient treatment with combined immunotherapy. He was started on nivolumab/relatlimab and received 3 doses a month apart. Unfortunately, the patient expired within 6 months after being diagnosed with malignant melanoma.

## DISCUSSION

Melanocytes are the melanin-carrying cells of the basal and suprabasal layer of the epidermis that are derived by melanogenesis from neural crest cells.^4^ This process is underpinned by multiple events at the molecular level that are susceptible to mutations in the respective DNA segments, leading to formation of melanoma.^5^ A dose-dependent relationship has been demonstrated between exposure to ultraviolet irradiation and development of melanoma; hence, the preponderance of malignant melanoma in ultraviolet exposed body areas such as the eye and skin.^6^ PMME are extremely rare, and traditional risk factors are thought to play a minimal role in their oncogenic process. Furthermore, PMME assumes varied morphological and histological characteristics and may be asymmetrical, irregularly shaped, with varied pigmentation.^1^

It is estimated that just about 480 cases of PMME have been reported since the first case was described by Baur in 1906, with >90% located in the middle and lower thirds of the esophagus.^7^ There are no clearly defined environmental exposures that are associated with PMME, with only indirect causality attributed to smoking cigarettes in some of the cases. PMMEs are thought to be twice as common in male patients as in female patients, occurring mostly in the 6th and 7th decade of life. The higher prevalence in older patients seems to point to the existence of early gene mutations that accrue slowly over the life of an individual in traditionally noncancerous non-UV exposed cells, leading to PMME.^8^

Approximately 80% of patients with PMME present with dysphagia and chest pain often leading to upper gastrointestinal endoscopic examination with the discovery and biopsy of a pigmented mass.^9^ While a black pigmented lesion on EGD for our patient was consistent with a diagnostic criterion outlined by Allen and Spitz (1953) for suspected melanoma, evidence shows that up to 10%–25% of PMME cases are amelanotic and colorless.^10,11^ Therefore, immunohistochemical staining techniques and genetic analysis have become critical to the accurate diagnosis and prognostication of PMME.

The MART-1 and HMB-45 are the most specific antibodies available to differentiate melanomas from other mucosal tumors; hence, the presence of MART-1 on the immunohistochemical panel of our patient confirmed the diagnosis of malignant melanoma.^12^ This helped rule out other neoplasms, including Kaposi sarcoma, esophageal lymphoma, and carcinoma. Even then, it is necessary to confirm that a gastrointestinal melanoma lesion is not a result of metastatic spread from a primary cutaneous or other mucosal site. While demonstration of melanin in tumor and adjacent tissue would be helpful, this would miss out on the significant number of malignant melanomas that are amelanocytic.^11^ It is therefore imperative for providers to conduct a thorough physical examination to ensure that there are no other melanoma lesions on skin and other mucosal surfaces. Thus, a diagnosis of PMME in our patient was made due to a lack of physical evidence of other potential primary lesions, a positive MART-1 antigen and negative cytokeratin antigen on immunohistochemical testing.^13^ This was further confirmed by positron emission tomography-computed tomography showing evidence of large mass with increased activity extending from the mid-to-distal esophagus and lack of similar activity in any other part of the body.

The mainstay of treatment for localized PMME is surgical excision.^14^ However, this was not an option in our patient due to the large size of the tumor extending from distal esophagus to gastroesophageal junction and metastatic spread to paraoesophageal and gastrohepatic ligament, and upper surface of the liver as shown on positron emission tomography. Thus, the only treatment option for this unresectable/locally advanced disease such as in our patient was the initiation of chemotherapy or immunotherapy. The treating physician elected combination immunotherapy (nivolumab/relatlimab), based on available evidence of progression-free survival of 15.6 months with immunotherapy compared with 3 months for chemotherapy only.^11^

Currently, there is no evidence confirming if our patient would have benefitted from a combination regimen comprising both chemotherapy and immunotherapy in any specific sequence. Indeed, some patients who have experienced disease progression on immunotherapy have demonstrated robust responses to salvage chemotherapy in a synergistic fashion.^15^ This is thought to be due to the ability of immunotherapy to activate CD8 T-cell response which in turn increases CD69 and PD1 expression in malignant melanoma, thereby enhancing clinical response to chemotherapy.^15^ There is, however, need for more studies to investigate this synergy for future patients with PMME.

## DISCLOSURES

Author contributions: A. Chaudhry, G. Buluku, C. Suryanarayana, and J. Noor wrote the discussion and case report sections. M. Chaudhari and S. Bigler wrote the pathology slides. S. Boyd and M. Lee edited the drafts. A. Chaudhry is the article guarantor.

Financial disclosure: None to report.

Informed consent was obtained for this case report.
